# Supplementary material for: Revolutionizing CAD/CAM-based restorative dental processes and materials with artificial intelligence: a concise narrative review
Source: PeerJ. 2024 Jul 19;12:e17793. doi: 10.7717/peerj.17793 (PMC11262301; doi:10.7717/peerj.17793)
Supplement: Supplemental Information 1 [file peerj-12-17793-s001.docx]

**SEARCH STRATEGY FOR THE LITERATURE REVIEW:**

| Objective: | -The objective of this survey is to provide a concise and simplified overview of the most recent dental CAD/CAM and AI advancements for readers with dental backgrounds, rather than a detailed report and appraisal of the available literature.  -The survey aims to conduct a non-exhaustive search of the literature from January 2000 to January 2024 and present the results in a narrative format without any quantitative data synthesis. | | | |
| --- | --- | --- | --- | --- |
| Search Strategy: | | | | |
| 1. Databases: | - PubMed/Medline | - Web of Science | | - Google Scholar |
| 2. Search Terms:  - "Artificial Intelligence"  - "CAD/CAM"  - "Machine Learning"  - "Digital Dentistry"  - "Neural Networks"  - "Virtual Reality" - "Restorative Dentistry"  - “Natural Language Processing”  -“Fuzzy Logic" | Search using Boolean operators: | | | |
|  | (“CAD/CAM” [MeSH] OR “digital dentistry" [MeSH] or “digital restorative dentistry” [MeSH] OR “computer aided design computer aided milling” [MeSH] or “restorative dentistry” AND (“Artificial intelligence" [MeSH] OR “Machine Learning” OR “Virtual Reallity” [MeSH] OR “AI-based” OR “Deep learning" [MeSH] OR “Fuzzy Logic" [MeSH] OR “Natural Language Processing" [MeSH] OR “Neural Networks" [MeSH])  Results (93 records) | (“CAD/CAM” OR “digital dentistry” OR “digital restorative dentistry” OR “computer aided design computer aided milling” OR “restorative dentistry”) AND (“Artificial intelligence” OR “Machine Learning” OR “Virtual Reallity” OR “AI-based” OR “Fuzzy Logic” OR “Natural Language Processing” OR “Neural Networks” NOT (MEDLINE)  Results (159 records) | (“CAD/CAM” OR “digital dentistry” OR “digital restorative dentistry” OR “computer aided design computer aided milling” OR “restorative dentistry”) AND (“Artificial intelligence” OR “Machine Learning” OR “Virtual Reallity” OR “AI-based” OR “Fuzzy Logic” OR “Natural Language Processing” OR “Neural Networks”)  Results (203 records) | |
| 3. Inclusion Criteria: | - Articles written in English  - Published between January 2000 and January 2024  - Relevant to restorative dental CAD/CAM and AI advancements | | | |
| 4. Exclusion Criteria: | - Conference proceedings, abstracts, and commentaries  - Articles primarily discussing industrial or medical CAD/CAM  - Articles related to other dental specialties  -Articles related to restorative dentistry without the use of CAD/CAM | | | |
| 5. Search Method: | - Employed a free-text protocol with combinations of the specified keywords (and MeSH)  - Conducted a thorough search within article references and of recently advertised dental AI applications from various manufacturers | | | |
| Selection Process:  (The screening software, Rayyan, developed by the Qatar Computing Research Institute (QCRI), was used in the screening and deduplication process.) Two reviewers conducted the screening, and conflict was resolved by discussion. | | | | |
| 1. Initial Screening: | - Titles and abstracts of the retrieved articles were screened to assess relevance to the survey objective. | | | |
| 2. Full-Text Review: | - Articles passing the initial screening underwent a full-text review for eligibility. | | | |
| Data Extraction: | - Relevant data from the selected articles were extracted, including details on dental CAD/CAM and AI advancements. | | | |
| Reporting: | - The results of the search were reported in a narrative format without any quantitative data synthesis. | | | |
| Total number of retrieved abstracts after duplicates removal: | 395 (89 duplicates) (duplicates removed using Rayyan online literature review software.). from those most relevant articles were further screened to remove non-relevant papers per the inclusion and exclusion criteria and reviewers’ discussion) | | | |
| Articles included in this narrative literature review following inclusion and exclusion criteria and relevance: | 61 articles relative to : Artificial Intelligence integration into restorative dental CAD/CAM systems and AI applications for dental CAD/CAM materials. | | | |

**Papers included in the narrative literature review per the search strategy employed:**

1. Thurzo A, Urbanová W, Novák B, Czako L, Siebert T, Stano P, Mareková S, Fountoulaki G, Kosnáčová H, Varga I: **Where Is the Artificial Intelligence Applied in Dentistry? Systematic Review and Literature Analysis**. *Healthcare* 2022, **10**(7):1269.

2. Alqutaibi AY, Aboalrejal AN: **ARTIFICIAL INTELLIGENCE (AI) AS AN AID IN RESTORATIVE DENTISTRY IS PROMISING, BUT STILL A WORK IN PROGRESS**. *J Evid Based Dent Pract* 2023, **23**(1):101837.

3. Schwendicke F, Cejudo Grano de Oro J, Garcia Cantu A, Meyer-Lueckel H, Chaurasia A, Krois J: **Artificial Intelligence for Caries Detection: Value of Data and Information**. *J Dent Res* 2022, **101**(11):1350-1356.

4. Lin GSS, Ng YS, Ghani N, Chua KH: **Revolutionising dental technologies: a qualitative study on dental technicians' perceptions of Artificial intelligence integration**. *BMC Oral Health* 2023, **23**(1):690.

5. Naidu V, Jaju S: **CAD/CAM Engineering And Artificial Intelligence In Dentistry**. *IOP Conference Series: Materials Science and Engineering* 2022, **1259**(1):012018.

6. Ding H, Wu J, Zhao W, Matinlinna JP, Burrow MF, Tsoi JKH: **Artificial intelligence in dentistry—A review**. *Frontiers in Dental Medicine* 2023, **4**.

7. Mushtaq TB, Azam S, Baig I: **Artificial Intelligence in Dentistry: Literature Review**. *Journal of Pharmaceutical Research International* 2022, **34**(53B):7-14.

8. Ng KW, Poynting S: **Artificial Intelligence in CAD/CAM**; 1995.

9. Upmanyu S, Upmanyu A, Jamwal A, Agrawal R: **Machine Learning in CAD/CAM: What We Think We Know So Far and What We Don’t**. In: *Recent Advances in Industrial Production: 2022// 2022; Singapore*: Springer Singapore; 2022: 495-507.

10. Susic I, Travar M, Susic M: **The application of CAD / CAM technology in Dentistry**. *IOP Conference Series: Materials Science and Engineering* 2017, **200**:012020.

11. Regassa Hunde B, Debebe Woldeyohannes A: **Future prospects of computer-aided design (CAD) – A review from the perspective of artificial intelligence (AI), extended reality, and 3D printing**. *Results in Engineering* 2022, **14**:100478.

12. Leeson D: **The digital factory in both the modern dental lab and clinic**. *Dent Mater* 2020, **36**(1):43-52.

13. Li H, Sakai T, Tanaka A, Ogura M, Lee C, Yamaguchi S, Imazato S: **Interpretable AI Explores Effective Components of CAD/CAM Resin Composites**. *Journal of Dental Research* 2022, **101**(11):1363-1371.

14. Barredo Arrieta A, Díaz-Rodríguez N, Del Ser J, Bennetot A, Tabik S, Barbado A, Garcia S, Gil-Lopez S, Molina D, Benjamins R *et al*: **Explainable Artificial Intelligence (XAI): Concepts, taxonomies, opportunities and challenges toward responsible AI**. *Information Fusion* 2020, **58**:82-115.

15. Gurel G: **The Artificial Intelligence-Based 3D Smile Design: REBEL**. In: *Esthetic Oral Rehabilitation with Veneers: A Guide to Treatment Preparation and Clinical Concepts.* edn. Edited by Trushkowsky RD. Cham: Springer International Publishing; 2020: 235-263.

16. Blatz MB, Chiche G, Bahat O, Roblee R, Coachman C, Heymann HO: **Evolution of Aesthetic Dentistry**. *J Dent Res* 2019, **98**(12):1294-1304.

17. Alikhasi M, Yousefi P, Afrashtehfar KI: **Smile Design: Mechanical Considerations**. *Dent Clin North Am* 2022, **66**(3):477-487.

18. Grischke J, Johannsmeier L, Eich L, Griga L, Haddadin S: **Dentronics: Towards robotics and artificial intelligence in dentistry**. *Dental Materials* 2020, **36**(6):765-778.

19. Wang F, Tang Q, Xi S, Liu R, Niu L: **Comparison and evaluation of the morphology of crowns generated by biogeneric design technique with CEREC chairside system**. *PLoS One* 2020, **15**(1):e0227050.

20. Saleh O, Nozaki K, Matsumura M, Yanaka W, Abdou A, Miura H, Fueki K: **Emergence angle: Comprehensive analysis and machine learning prediction for clinical application**. *J Prosthodont Res* 2022.

21. Oldhoff MGE, Mirzaali MJ, Tümer N, Zhou J, Zadpoor AA: **Comparison in clinical performance of surgical guides for mandibular surgery and temporomandibular joint implants fabricated by additive manufacturing techniques**. *Journal of the Mechanical Behavior of Biomedical Materials* 2021, **119**:104512.

22. Farook TH, Jamayet NB, Abdullah JY, Asif JA, Rajion ZA, Alam MK: **Designing 3D prosthetic templates for maxillofacial defect rehabilitation: A comparative analysis of different virtual workflows**. *Comput Biol Med* 2020, **118**:103646.

23. Koralakunte PR, Aljanakh M: **The role of virtual articulator in prosthetic and restorative dentistry**. *J Clin Diagn Res* 2014, **8**(7):Ze25-28.

24. Luthra RP, Gupta R, Kumar N, Mehta S, Sirohi R: **VIRTUAL ARTICULATORS IN PROSTHETIC DENTISTRY : A REVIEW**. In*: 2015*; 2015.

25. Coachman C, Georg R, Bohner L, Rigo LC, Sesma N: **Chairside 3D digital design and trial restoration workflow**. *J Prosthet Dent* 2020, **124**(5):514-520.

26. Revilla-León M, Kois DE, Zeitler JM, Att W, Kois JC: **An overview of the digital occlusion technologies: Intraoral scanners, jaw tracking systems, and computerized occlusal analysis devices**. *J Esthet Restor Dent* 2023, **35**(5):735-744.

27. Schlenz MA, Schupp B, Schmidt A, Wöstmann B, Baresel I, Krämer N, Schulz-Weidner N: **New Caries Diagnostic Tools in Intraoral Scanners: A Comparative In Vitro Study to Established Methods in Permanent and Primary Teeth**. *Sensors* 2022, **22**(6).

28. Moran MBH, Faria MDB, Bastos LF, Giraldi GA, Conci A: **Combining Image Processing and Artificial Intelligence for Dental Image Analysis: Trends, Challenges, and Applications**. In: *EAI/Springer Innovations in Communication and Computing.* 2022: 75-105.

29. Brown C: **Artificial Intelligence in Dentistry Not just science fiction anymore**. *Inside Dental Technology* 2019, **10**(7).

30. Tran M: **AI Isn't Science Fiction: It's Digital Design's Reality Deep learning proves effective in designing crowns**. *Inside Dental Technology* 2022, **12**(7).

31. Zhang C, Fan L, Zhang S, Zhao J, Gu Y: **Deep learning based dental implant failure prediction from periapical and panoramic films**. *Quantitative Imaging in Medicine and Surgery* 2023, **13**(2):935-945.

32. Hashem M, Mohammed ML, Youssef AE: **Improving the Efficiency of Dental Implantation Process Using Guided Local Search Models and Continuous Time Neural Networks with Robotic Assistance**. *IEEE Access* 2020, **8**:202755-202764.

33. Schwendicke Fa, Samek W, Krois J: **Artificial intelligence in dentistry: chances and challenges**. *Journal of dental research* 2020, **99**(7):769-774.

34. Roongruangsilp P, Khongkhunthian P: **Artificial intelligence with the application in medicine and dentistry**. *JOURNAL OF OSSEOINTEGRATION* 2022, **14**(3).

35. Revilla-León M, Gómez-Polo M, Vyas S, Barmak BA, Gallucci GO, Att W, Özcan M, Krishnamurthy VR: **Artificial intelligence models for tooth-supported fixed and removable prosthodontics: A systematic review**. *J Prosthet Dent* 2021.

36. Mangano FG, Admakin O, Lerner H, Mangano C: **Artificial intelligence and augmented reality for guided implant surgery planning: A proof of concept**. *J Dent* 2023, **133**:104485.

37. Giglio GD, Giglio AB, Tarnow DP: **A Paradigm Shift Using Scan Bodies to Record the Position of a Complete Arch of Implants in a Digital Workflow**. *Int J Periodontics Restorative Dent* 2024, **44**(1):115-126.

38. Imagoworks: **Imagoworks Launches AI-Based Web Dental CAD 3Dme Crown**. *Dentistry Today* 2022.

39. Mitali Pareek BK: **Artificial intelligence in prosthodontics: a scoping review on current applications and future possibilities.** *International Journal of Advances in Medicine* 2022, **9**(3):3.

40. Lerner H, Mouhyi J, Admakin O, Mangano F: **Artificial intelligence in fixed implant prosthodontics: a retrospective study of 106 implant-supported monolithic zirconia crowns inserted in the posterior jaws of 90 patients**. *BMC Oral Health* 2020, **20**(1):80.

41. Cho JH, Çakmak G, Yi Y, Yoon HI, Yilmaz B, Schimmel M: **Tooth morphology, internal fit, occlusion and proximal contacts of dental crowns designed by deep learning-based dental software: A comparative study**. *J Dent* 2024, **141**:104830.

42. Liu CM, Lin WC, Lee SY: **Evaluation of the efficiency, trueness, and clinical application of novel artificial intelligence design for dental crown prostheses**. *Dent Mater* 2024, **40**(1):19-27.

43. Tian S, Wang M, Dai N, Ma H, Li L, Fiorenza L, Sun Y, Li Y: **DCPR-GAN: Dental Crown Prosthesis Restoration Using Two-Stage Generative Adversarial Networks**. *IEEE Journal of Biomedical and Health Informatics* 2022, **26**(1):151-160.

44. Tian S, Wang M, Dai N, Ma H, Li L, Fiorenza L, Sun Y, Li Y: **DCPR-GAN: dental crown prosthesis restoration using two-stage generative adversarial networks**. *IEEE Journal of Biomedical and Health Informatics* 2021, **26**(1):151-160.

45. Tian S, Wang M, Ma H, Huang P, Dai N, Sun Y, Meng J: **Efficient tooth gingival margin line reconstruction via adversarial learning**. *Biomedical Signal Processing and Control* 2022, **78**:103954.

46. Chau RCW, Hsung RT, McGrath C, Pow EHN, Lam WYH: **Accuracy of artificial intelligence-designed single-molar dental prostheses: A feasibility study**. *J Prosthet Dent* 2023.

47. Ding H, Cui Z, Maghami E, Chen Y, Matinlinna JP, Pow EHN, Fok ASL, Burrow MF, Wang W, Tsoi JKH: **Morphology and mechanical performance of dental crown designed by 3D-DCGAN**. *Dental Materials* 2023, **39**(3):320-332.

48. Farook TH, Ahmed S, Jamayet NB, Rashid F, Barman A, Sidhu P, Patil P, Lisan AM, Eusufzai SZ, Dudley J *et al*: **Computer-aided design and 3-dimensional artificial/convolutional neural network for digital partial dental crown synthesis and validation**. *Scientific Reports* 2023, **13**(1).

49. CIMsystem: **Efficiency and automation with AI DRIVEN, Artificial Intelligence applied to dental manufacturing.** In*.*; 2021.

50. Bernauer SA, Zitzmann NU, Joda T: **The use and performance of artificial intelligence in prosthodontics: A systematic review**. *Sensors* 2021, **21**(19).

51. Yamaguchi S, Lee C, Karaer O, Ban S, Mine A, Imazato S: **Predicting the Debonding of CAD/CAM Composite Resin Crowns with AI**. *J Dent Res* 2019, **98**(11):1234-1238.

52. Kose C, Jr., Oliveira D, Pereira PNR, Rocha MG: **Using artificial intelligence to predict the final color of leucite-reinforced ceramic restorations**. *J Esthet Restor Dent* 2023.

53. Gur E, Mendlovic D, Zalevsky Z: **Optical implementation of fuzzy-logic controllers. Part II**. *Applied Optics* 1999, **38**(20):4354-4358.

54. Herrera L, Pulgar R, Santana J, Cardona J, Guillén A, Rojas I, Pérez Gómez MdM: **Prediction of color change after tooth bleaching using fuzzy logic for Vita Classical shades identification**. *Applied optics* 2010, **49**:422-429.

55. Butler KT, Davies DW, Cartwright H, Isayev O, Walsh A: **Machine learning for molecular and materials science**. *Nature* 2018, **559**(7715):547-555.

56. Chen C-T, Gu GX: **Machine learning for composite materials**. *MRs Communications* 2019, **9**(2):556-566.

57. Ward L, Agrawal A, Choudhary A, Wolverton C: **A general-purpose machine learning framework for predicting properties of inorganic materials**. *npj Computational Materials* 2016, **2**(1):16028.

58. Grymak A, Tieh MT, Yang AHX, Choi JJE: **Development of predictive algorithms for the wear resistance of denture teeth materials**. *J Mech Behav Biomed Mater* 2023, **144**:105984.

59. Babu A, Onesimu A, Sagayam M: **Artificial Intelligence in dentistry: Concepts, Applications and Research Challenges**. *E3S Web of Conferences* 2021, **297**:01074.

60. Basu B, Gowtham NH, Xiao Y, Kalidindi SR, Leong KW: **Biomaterialomics: Data science-driven pathways to develop fourth-generation biomaterials**. *Acta Biomater* 2022, **143**:1-25.

61. Shriya R. Singi SS, Amit R. Reche, Akash Sibal, Namrata Mantri: **Extended Arm of Precision in Prosthodontics: Artificial Intelligence.** . *Cureus* 2022, **14**(11):e30962.

**References Included in the full paper:**

1. Abdullah A, Muhammed F, Zheng B, and Liu Y. 2018. An Overview of Computer Aided Design/Computer Aided Manufacturing (CAD/CAM) in Restorative Dentistry. 7:1-10. 10.22038/jdmt.2017.26351.1213
2. Abduo J, Lyons K, and Bennamoun M. 2014. Trends in computer-aided manufacturing in prosthodontics: a review of the available streams. *Int J Dent* 2014:783948. 10.1155/2014/783948
3. Aijaz SF, Khan SJ, Azim F, Shakeel CS, and Hassan U. 2022. Deep Learning Application for Effective Classification of Different Types of Psoriasis. *Journal of Healthcare Engineering* 2022. 10.1155/2022/7541583
4. Alauddin MS, Baharuddin AS, and Ghazali MIM. 2021. The modern and digital transformation of oral health care: A mini review. *Healthcare (Switzerland)* 9. 10.3390/healthcare9020118
5. Alghazzawi TF. 2016. Advancements in CAD/CAM technology: Options for practical implementation. *Journal of Prosthodontic Research* 60 2:72-84.
6. Alikhasi M, Yousefi P, and Afrashtehfar KI. 2022. Smile Design: Mechanical Considerations. *Dent Clin North Am* 66:477-487. 10.1016/j.cden.2022.02.008
7. Alqutaibi AY, and Aboalrejal AN. 2023. ARTIFICIAL INTELLIGENCE (AI) AS AN AID IN RESTORATIVE DENTISTRY IS PROMISING, BUT STILL A WORK IN PROGRESS. *J Evid Based Dent Pract* 23:101837. 10.1016/j.jebdp.2023.101837
8. Alzahrani SJ, Hajjaj MS, Azhari AA, Ahmed WM, Yeslam HE, and Carvalho RM. 2023. Mechanical Properties of Three-Dimensional Printed Provisional Resin Materials for Crown and Fixed Dental Prosthesis: A Systematic Review. *Bioengineering* 10:663.
9. Babu A, Onesimu A, and Sagayam M. 2021. Artificial Intelligence in dentistry: Concepts, Applications and Research Challenges. *E3S Web of Conferences* 297:01074. 10.1051/e3sconf/202129701074
10. Barredo Arrieta A, Díaz-Rodríguez N, Del Ser J, Bennetot A, Tabik S, Barbado A, Garcia S, Gil-Lopez S, Molina D, Benjamins R, Chatila R, and Herrera F. 2020. Explainable Artificial Intelligence (XAI): Concepts, taxonomies, opportunities and challenges toward responsible AI. *Information Fusion* 58:82-115. <https://doi.org/10.1016/j.inffus.2019.12.012>
11. Basheer IA, and Hajmeer M. 2000. Artificial neural networks: fundamentals, computing, design, and application. *Journal of microbiological methods* 43:3-31.
12. Basu B, Gowtham NH, Xiao Y, Kalidindi SR, and Leong KW. 2022. Biomaterialomics: Data science-driven pathways to develop fourth-generation biomaterials. *Acta Biomater* 143:1-25. 10.1016/j.actbio.2022.02.027
13. Bernauer SA, Zitzmann NU, and Joda T. 2021. The use and performance of artificial intelligence in prosthodontics: A systematic review. *Sensors* 21. 10.3390/s21196628
14. Blatz MB, Chiche G, Bahat O, Roblee R, Coachman C, and Heymann HO. 2019. Evolution of Aesthetic Dentistry. *J Dent Res* 98:1294-1304. 10.1177/0022034519875450
15. Brown C. 2019. Artificial Intelligence in Dentistry Not just science fiction anymore. *Inside Dental Technology* 10.
16. Butler KT, Davies DW, Cartwright H, Isayev O, and Walsh A. 2018. Machine learning for molecular and materials science. *Nature* 559:547-555.
17. Carleo G, Cirac I, Cranmer K, Daudet L, Schuld M, Tishby N, Vogt-Maranto L, and Zdeborová L. 2019. Machine learning and the physical sciences. *Reviews of Modern Physics* 91:045002.
18. Carrillo-Perez F, Pecho OE, Morales JC, Paravina RD, Della Bona A, Ghinea R, Pulgar R, Pérez MDM, and Herrera LJ. 2022. Applications of artificial intelligence in dentistry: A comprehensive review. *Journal of Esthetic and Restorative Dentistry* 34:259-280. 10.1111/jerd.12844
19. Chau RCW, Hsung RT, McGrath C, Pow EHN, and Lam WYH. 2023. Accuracy of artificial intelligence-designed single-molar dental prostheses: A feasibility study. *J Prosthet Dent*. 10.1016/j.prosdent.2022.12.004
20. Chen C-T, and Gu GX. 2019. Machine learning for composite materials. *MRs Communications* 9:556-566.
21. Cho JH, Çakmak G, Yi Y, Yoon HI, Yilmaz B, and Schimmel M. 2024. Tooth morphology, internal fit, occlusion and proximal contacts of dental crowns designed by deep learning-based dental software: A comparative study. *J Dent* 141:104830. 10.1016/j.jdent.2023.104830
22. CIMsystem. 2021. Efficiency and automation with AI DRIVEN, Artificial Intelligence applied to dental manufacturing.
23. Coachman C, Georg R, Bohner L, Rigo LC, and Sesma N. 2020. Chairside 3D digital design and trial restoration workflow. *J Prosthet Dent* 124:514-520. 10.1016/j.prosdent.2019.10.015
24. Deng G, Zhu J, Lu Q, Liu C, Liang T, Jiang J, Li H, Zhou C, Wu S, Chen T, Chen J, Yao Y, Liao S, Yu C, Huang S, Sun X, Chen L, Ye Z, Guo H, Chen W, Jiang W, Fan B, Yang Z, Gu W, Wang Y, and Zhan X. 2023. Application of machine learning in prediction of bone cement leakage during single-level thoracolumbar percutaneous vertebroplasty. *BMC Surgery* 23. 10.1186/s12893-023-01959-y
25. Ding H, Cui Z, Maghami E, Chen Y, Matinlinna JP, Pow EHN, Fok ASL, Burrow MF, Wang W, and Tsoi JKH. 2023a. Morphology and mechanical performance of dental crown designed by 3D-DCGAN. *Dental Materials* 39:320-332. <https://doi.org/10.1016/j.dental.2023.02.001>
26. Ding H, Wu J, Zhao W, Matinlinna JP, Burrow MF, and Tsoi JKH. 2023b. Artificial intelligence in dentistry—A review. *Frontiers in Dental Medicine* 4. 10.3389/fdmed.2023.1085251
27. Duret F. 1991. [Dental CAD-CAM six years after the first presentation at the 1985 A.D.F. Congress]. *Actual Odontostomatol (Paris)* 45:431-454.
28. Fang G, Chow MC, Ho JD, He Z, Wang K, Ng T, Tsoi JK, Chan P-L, Chang H-C, and Chan DT-M. 2021. Soft robotic manipulator for intraoperative MRI-guided transoral laser microsurgery. *Science Robotics* 6:eabg5575.
29. Farook TH, Ahmed S, Jamayet NB, Rashid F, Barman A, Sidhu P, Patil P, Lisan AM, Eusufzai SZ, Dudley J, and Daood U. 2023. Computer-aided design and 3-dimensional artificial/convolutional neural network for digital partial dental crown synthesis and validation. *Scientific Reports* 13. 10.1038/s41598-023-28442-1
30. Farook TH, Jamayet NB, Abdullah JY, Asif JA, Rajion ZA, and Alam MK. 2020. Designing 3D prosthetic templates for maxillofacial defect rehabilitation: A comparative analysis of different virtual workflows. *Comput Biol Med* 118:103646. 10.1016/j.compbiomed.2020.103646
31. Fatima A, Shafi I, Afzal H, Díez IDLT, Lourdes DRSM, Breñosa J, Espinosa JCM, and Ashraf I. 2022a. Advancements in Dentistry with Artificial Intelligence: Current Clinical Applications and Future Perspectives. *Healthcare (Switzerland)* 10. 10.3390/healthcare10112188
32. Fatima A, Shafi I, Afzal H, Díez IT, Lourdes DRM, Breñosa J, Espinosa JCM, and Ashraf I. 2022b. Advancements in Dentistry with Artificial Intelligence: Current Clinical Applications and Future Perspectives. *Healthcare (Basel)* 10. 10.3390/healthcare10112188
33. Fatima A, Shafi I, Afzal H, Mahmood K, Díez IDLT, Lipari V, Ballester JB, and Ashraf I. 2023. Deep Learning-Based Multiclass Instance Segmentation for Dental Lesion Detection. *Healthcare (Switzerland)* 11. 10.3390/healthcare11030347
34. Feng Z, Dong Y, Zhao Y, Bai S, Zhou B, Bi Y, and Wu G. 2010. Computer-assisted technique for the design and manufacture of realistic facial prostheses. *Br J Oral Maxillofac Surg* 48:105-109. 10.1016/j.bjoms.2009.05.009
35. Giglio GD, Giglio AB, and Tarnow DP. 2024. A Paradigm Shift Using Scan Bodies to Record the Position of a Complete Arch of Implants in a Digital Workflow. *Int J Periodontics Restorative Dent* 44:115-126. 10.11607/prd.6733
36. Grischke J, Johannsmeier L, Eich L, Griga L, and Haddadin S. 2020. Dentronics: Towards robotics and artificial intelligence in dentistry. *Dental Materials* 36:765-778.
37. Grymak A, Tieh MT, Yang AHX, and Choi JJE. 2023. Development of predictive algorithms for the wear resistance of denture teeth materials. *J Mech Behav Biomed Mater* 144:105984. 10.1016/j.jmbbm.2023.105984
38. Grzebieluch W, Kowalewski P, Sopel M, and Mikulewicz M. 2022. Influence of Artificial Aging on Mechanical Properties of Six Resin Composite Blocks for CAD/CAM Application. *Coatings* 12:837.
39. Gur E, Mendlovic D, and Zalevsky Z. 1999. Optical implementation of fuzzy-logic controllers. Part II. *Applied optics* 38:4354-4358. 10.1364/AO.38.004354
40. Gurel G. 2020. The Artificial Intelligence-Based 3D Smile Design: REBEL. In: Trushkowsky RD, ed. *Esthetic Oral Rehabilitation with Veneers: A Guide to Treatment Preparation and Clinical Concepts*. Cham: Springer International Publishing, 235-263.
41. Güth JF, Runkel C, Beuer F, Stimmelmayr M, Edelhoff D, and Keul C. 2017. Accuracy of five intraoral scanners compared to indirect digitalization. *Clin Oral Investig* 21:1445-1455. 10.1007/s00784-016-1902-4
42. Hajjaj MS, Alamoudi RAA, Babeer WA, Rizg WY, Basalah AA, Alzahrani SJ, and Yeslam HE. 2024. Flexural strength, flexural modulus and microhardness of milled vs. fused deposition modeling printed Zirconia; effect of conventional vs. speed sintering. *BMC Oral Health* 24:38. 10.1186/s12903-023-03829-8
43. Hashem M, Mohammed ML, and Youssef AE. 2020. Improving the Efficiency of Dental Implantation Process Using Guided Local Search Models and Continuous Time Neural Networks with Robotic Assistance. *IEEE Access* 8:202755-202764. 10.1109/ACCESS.2020.3034689
44. Herrera L, Pulgar R, Santana J, Cardona J, Guillén A, Rojas I, and Pérez Gómez MdM. 2010. Prediction of color change after tooth bleaching using fuzzy logic for Vita Classical shades identification. *Applied optics* 49:422-429. 10.1364/AO.49.000422
45. Imagoworks. 2022. Imagoworks Launches AI-Based Web Dental CAD 3Dme Crown. *Dentistry Today*.
46. Jain R, Takkar R, Jain GC, Takkar R, and Deora N. 2016. CAD-CAM the future of digital dentistry: a review.
47. Jiao T, Zhang F, Huang X, and Wang C. 2004. Design and fabrication of auricular prostheses by CAD/CAM system. *Int J Prosthodont* 17:460-463.
48. Joda T, Waltimo T, Pauli-Magnus C, Probst-Hensch N, and Zitzmann NU. 2018. Population-based linkage of big data in dental research. *International Journal of Environmental Research and Public Health* 15:2357.
49. Khan RA, Luo Y, and Wu FX. 2022. Machine learning based liver disease diagnosis: A systematic review. *Neurocomputing* 468:492-509. 10.1016/j.neucom.2021.08.138
50. Khanagar SB, Alfouzan K, Alkadi L, Albalawi F, Iyer K, and Awawdeh M. 2022a. Performance of Artificial Intelligence (AI) Models Designed for Application in Pediatric Dentistry—A Systematic Review. *Applied Sciences* 12:9819.
51. Khanagar SB, Alfouzan K, Awawdeh M, Alkadi L, Albalawi F, and Alfadley A. 2022b. Application and Performance of Artificial Intelligence Technology in Detection, Diagnosis and Prediction of Dental Caries (DC)-A Systematic Review. *Diagnostics (Basel)* 12. 10.3390/diagnostics12051083
52. Koralakunte PR, and Aljanakh M. 2014. The role of virtual articulator in prosthetic and restorative dentistry. *Journal of clinical and diagnostic research : JCDR* 8:Ze25-28. 10.7860/jcdr/2014/8929.4648
53. Kose C, Jr., Oliveira D, Pereira PNR, and Rocha MG. 2023. Using artificial intelligence to predict the final color of leucite-reinforced ceramic restorations. *J Esthet Restor Dent*. 10.1111/jerd.13007
54. Kulin M, Kazaz T, Moerman I, and De Poorter E. 2020. *A survey on Machine Learning-based Performance Improvement of Wireless Networks: PHY, MAC and network layer*.
55. Leeson D. 2020. The digital factory in both the modern dental lab and clinic. *Dent Mater* 36:43-52. 10.1016/j.dental.2019.10.010
56. Lepidi L, Galli M, Mastrangelo F, Venezia P, Joda T, Wang HL, and Li J. 2021. Virtual Articulators and Virtual Mounting Procedures: Where Do We Stand? *J Prosthodont* 30:24-35. 10.1111/jopr.13240
57. Lerner H, Mouhyi J, Admakin O, and Mangano F. 2020. Artificial intelligence in fixed implant prosthodontics: a retrospective study of 106 implant-supported monolithic zirconia crowns inserted in the posterior jaws of 90 patients. *BMC Oral Health* 20:80. 10.1186/s12903-020-1062-4
58. Li H, Sakai T, Tanaka A, Ogura M, Lee C, Yamaguchi S, and Imazato S. 2022. Interpretable AI Explores Effective Components of CAD/CAM Resin Composites. *Journal of Dental Research* 101:1363-1371. 10.1177/00220345221089251
59. Lin GSS, Ng YS, Ghani N, and Chua KH. 2023. Revolutionising dental technologies: a qualitative study on dental technicians' perceptions of Artificial intelligence integration. *BMC Oral Health* 23:690. 10.1186/s12903-023-03389-x
60. Liu CM, Lin WC, and Lee SY. 2024. Evaluation of the efficiency, trueness, and clinical application of novel artificial intelligence design for dental crown prostheses. *Dent Mater* 40:19-27. 10.1016/j.dental.2023.10.013
61. Luthra RP, Gupta R, Kumar N, Mehta S, and Sirohi R. 2015. VIRTUAL ARTICULATORS IN PROSTHETIC DENTISTRY : A REVIEW.
62. Ma J, Schneider L, Lapuschkin S, Achtibat R, Duchrau M, Krois J, Schwendicke F, and Samek W. 2022. Towards Trustworthy AI in Dentistry. *J Dent Res* 101:1263-1268. 10.1177/00220345221106086
63. Mangano FG, Admakin O, Lerner H, and Mangano C. 2023. Artificial intelligence and augmented reality for guided implant surgery planning: A proof of concept. *J Dent* 133:104485. 10.1016/j.jdent.2023.104485
64. Maruta N, Morita K-i, Harazono Y, Anzai E, Akaike Y, Yamazaki K, Tonouchi E, and Yoda T. 2023. Automatic machine learning-based classification of mandibular third molar impaction status. *Journal of Oral and Maxillofacial Surgery, Medicine, and Pathology* 35:327-334. <https://doi.org/10.1016/j.ajoms.2022.12.010>
65. Mayta-Tovalino F, Munive-Degregori A, Luza S, Cárdenas-Mariño FC, Guerrero ME, and Barja-Ore J. 2023. Applications and Perspectives of Artificial Intelligence, Machine Learning and "Dentronics" in Dentistry: A Literature Review. *J Int Soc Prev Community Dent* 13:1-8. 10.4103/jispcd.JISPCD_35_22
66. Mitali Pareek BK. 2022. Artificial intelligence in prosthodontics: a scoping review on current applications and future possibilities. *International Journal of Advances in Medicine* 9:3. DOI: <https://dx.doi.org/10.18203/2349-3933.ijam20220444>
67. Miyazaki T, and Hotta Y. 2011. CAD/CAM systems available for the fabrication of crown and bridge restorations. *Aust Dent J* 56 Suppl 1:97-106. 10.1111/j.1834-7819.2010.01300.x
68. Mohammad-Rahimi H, Motamedian SR, Rohban MH, Krois J, Uribe SE, Mahmoudinia E, Rokhshad R, Nadimi M, and Schwendicke F. 2022. Deep learning for caries detection: A systematic review. *J Dent* 122:104115. 10.1016/j.jdent.2022.104115
69. Moran MBH, Faria MDB, Bastos LF, Giraldi GA, and Conci A. 2022. Combining Image Processing and Artificial Intelligence for Dental Image Analysis: Trends, Challenges, and Applications. EAI/Springer Innovations in Communication and Computing. p 75-105.
70. Mushtaq TB, Azam S, and Baig I. 2022. Artificial Intelligence in Dentistry: Literature Review. *JOURNAL OF PHARMACEUTICAL RESEARCH INTERNATIONAL* 34:7-14.
71. Naidu V, and Jaju S. 2022. CAD/CAM Engineering And Artificial Intelligence In Dentistry. *IOP Conference Series: Materials Science and Engineering* 1259:012018. 10.1088/1757-899X/1259/1/012018
72. Ng KW, and Poynting S. 1995. *Artificial Intelligence in CAD/CAM*.
73. Obermeyer Z, and Emanuel EJ. 2016. Predicting the Future - Big Data, Machine Learning, and Clinical Medicine. *N Engl J Med* 375:1216-1219. 10.1056/NEJMp1606181
74. Obermeyer Z, Powers B, Vogeli C, and Mullainathan S. 2019. Dissecting racial bias in an algorithm used to manage the health of populations. *Science* 366:447-453.
75. Oldhoff MGE, Mirzaali MJ, Tümer N, Zhou J, and Zadpoor AA. 2021. Comparison in clinical performance of surgical guides for mandibular surgery and temporomandibular joint implants fabricated by additive manufacturing techniques. *Journal of the Mechanical Behavior of Biomedical Materials* 119:104512. <https://doi.org/10.1016/j.jmbbm.2021.104512>
76. Oztekin F, Katar O, Sadak F, Yildirim M, Cakar H, Aydogan M, Ozpolat Z, Talo Yildirim T, Yildirim O, Faust O, and Acharya UR. 2023a. An Explainable Deep Learning Model to Prediction Dental Caries Using Panoramic Radiograph Images. *Diagnostics (Basel)* 13. 10.3390/diagnostics13020226
77. Oztekin F, Katar O, Sadak F, Yildirim M, Cakar H, Aydogan M, Ozpolat Z, Talo Yildirim T, Yildirim O, Faust O, and Acharya UR. 2023b. An Explainable Deep Learning Model to Prediction Dental Caries Using Panoramic Radiograph Images. *Diagnostics* 13:226. <https://dx.doi.org/10.3390/diagnostics13020226>
78. Page MJ, McKenzie JE, Bossuyt PM, Boutron I, Hoffmann TC, Mulrow CD, Shamseer L, Tetzlaff JM, Akl EA, Brennan SE, Chou R, Glanville J, Grimshaw JM, Hróbjartsson A, Lalu MM, Li T, Loder EW, Mayo-Wilson E, McDonald S, McGuinness LA, Stewart LA, Thomas J, Tricco AC, Welch VA, Whiting P, and Moher D. 2021. The PRISMA 2020 statement: an updated guideline for reporting systematic reviews. *BMJ* 372:n71. 10.1136/bmj.n71
79. Patil S, Albogami S, Hosmani J, Mujoo S, Kamil MA, Mansour MA, Abdul HN, Bhandi S, and Ahmed S. 2022. Artificial Intelligence in the Diagnosis of Oral Diseases: Applications and Pitfalls. *Diagnostics (Basel)* 12. 10.3390/diagnostics12051029
80. Prasad J, Mallikarjunaiah DR, Shetty A, Gandedkar N, Chikkamuniswamy AB, and Shivashankar PC. 2023. Machine Learning Predictive Model as Clinical Decision Support System in Orthodontic Treatment Planning. *Dentistry Journal* 11. 10.3390/dj11010001
81. Putra RH, Doi C, Yoda N, Astuti ER, and Sasaki K. 2022. Current applications and development of artificial intelligence for digital dental radiography. *Dento maxillo facial radiology* 51:20210197. 10.1259/dmfr.20210197
82. Qayyum A, Tahir A, Butt MA, Luke A, Abbas HT, Qadir J, Arshad K, Assaleh K, Imran MA, and Abbasi QH. 2023. Dental caries detection using a semi-supervised learning approach. *Sci Rep* 13:749. 10.1038/s41598-023-27808-9
83. Qu Y, Lin Z, Yang Z, Lin H, Huang X, and Gu L. 2022. Machine learning models for prognosis prediction in endodontic microsurgery. *Journal of Dentistry* 118. 10.1016/j.jdent.2022.103947
84. Regassa Hunde B, and Debebe Woldeyohannes A. 2022. Future prospects of computer-aided design (CAD) – A review from the perspective of artificial intelligence (AI), extended reality, and 3D printing. *Results in Engineering* 14:100478. <https://doi.org/10.1016/j.rineng.2022.100478>
85. Rekow ED. 2020. Digital dentistry: The new state of the art — Is it disruptive or destructive? *Dental Materials* 36:9-24. 10.1016/j.dental.2019.08.103
86. Revilla-Leon M, Gomez-Polo M, Vyas S, Barmak AB, Ozcan M, Att W, and Krishnamurthy VR. 2022. Artificial intelligence applications in restorative dentistry: A systematic review. *J Prosthet Dent* 128:867-875. 10.1016/j.prosdent.2021.02.010
87. Revilla-León M, Gómez-Polo M, Vyas S, Barmak BA, Gallucci GO, Att W, Özcan M, and Krishnamurthy VR. 2021. Artificial intelligence models for tooth-supported fixed and removable prosthodontics: A systematic review. *J Prosthet Dent*. 10.1016/j.prosdent.2021.06.001
88. Revilla-León M, Kois DE, Zeitler JM, Att W, and Kois JC. 2023. An overview of the digital occlusion technologies: Intraoral scanners, jaw tracking systems, and computerized occlusal analysis devices. *J Esthet Restor Dent* 35:735-744. 10.1111/jerd.13044
89. Reyes LT, Knorst JK, Ortiz FR, and Ardenghi TM. 2022. Machine Learning in the Diagnosis and Prognostic Prediction of Dental Caries: A Systematic Review. *Caries Research* 56:161-170. 10.1159/000524167
90. Roongruangsilp P, and Khongkhunthian P. 2022. Artificial intelligence with the application in medicine and dentistry. *JOURNAL OF OSSEOINTEGRATION* 14. 10.23805/JO.2022.14.22
91. Saleh O, Nozaki K, Matsumura M, Yanaka W, Abdou A, Miura H, and Fueki K. 2022. Emergence angle: Comprehensive analysis and machine learning prediction for clinical application. *J Prosthodont Res*. 10.2186/jpr.JPR_D_22_00194
92. Schlenz MA, Schupp B, Schmidt A, Wöstmann B, Baresel I, Krämer N, and Schulz-Weidner N. 2022. New Caries Diagnostic Tools in Intraoral Scanners: A Comparative In Vitro Study to Established Methods in Permanent and Primary Teeth. *Sensors* 22. 10.3390/s22062156
93. Schwendicke F, Cejudo Grano de Oro J, Garcia Cantu A, Meyer-Lueckel H, Chaurasia A, and Krois J. 2022a. Artificial Intelligence for Caries Detection: Value of Data and Information. *J Dent Res* 101:1350-1356. 10.1177/00220345221113756
94. Schwendicke F, Mertens S, Cantu AG, Chaurasia A, Meyer-Lueckel H, and Krois J. 2022b. Cost-effectiveness of AI for caries detection: randomized trial. *J Dent* 119:104080. 10.1016/j.jdent.2022.104080
95. Schwendicke Fa, Samek W, and Krois J. 2020. Artificial intelligence in dentistry: chances and challenges. *Journal of Dental Research* 99:769-774.
96. Shriya R. Singi SS, Amit R. Reche, Akash Sibal, Namrata Mantri. 2022. Extended Arm of Precision in Prosthodontics: Artificial Intelligence. . *Cureus* 14:e30962. doi:10.7759/cureus.30962
97. Spitznagel FA, Boldt J, and Gierthmuehlen PC. 2018. CAD/CAM Ceramic Restorative Materials for Natural Teeth. *J Dent Res* 97:1082-1091. 10.1177/0022034518779759
98. Susic I, Travar M, and Susic M. 2017. The application of CAD / CAM technology in Dentistry. *IOP Conference Series: Materials Science and Engineering* 200:012020. 10.1088/1757-899X/200/1/012020
99. Talpur S, Azim F, Rashid M, Syed SA, Talpur BA, and Khan SJ. 2022. Uses of Different Machine Learning Algorithms for Diagnosis of Dental Caries. *J Healthc Eng* 2022:5032435. 10.1155/2022/5032435
100. Thurzo A, Jančovičová V, Hain M, Thurzo M, Novák B, Kosnáčová H, Lehotská V, Varga I, Kováč P, and Moravanský N. 2022a. Human Remains Identification Using Micro-CT, Chemometric and AI Methods in Forensic Experimental Reconstruction of Dental Patterns after Concentrated Sulphuric Acid Significant Impact. *Molecules* 27:4035.
101. Thurzo A, Urbanová W, Novák B, Czako L, Siebert T, Stano P, Mareková S, Fountoulaki G, Kosnáčová H, and Varga I. 2022b. Where Is the Artificial Intelligence Applied in Dentistry? Systematic Review and Literature Analysis. *HEALTHCARE* 10:1269.
102. Tian S, Wang M, Dai N, Ma H, Li L, Fiorenza L, Sun Y, and Li Y. 2021. DCPR-GAN: dental crown prosthesis restoration using two-stage generative adversarial networks. *IEEE Journal of Biomedical and Health Informatics* 26:151-160.
103. Tian S, Wang M, Dai N, Ma H, Li L, Fiorenza L, Sun Y, and Li Y. 2022a. DCPR-GAN: Dental Crown Prosthesis Restoration Using Two-Stage Generative Adversarial Networks. *IEEE Journal of Biomedical and Health Informatics* 26:151-160. <https://dx.doi.org/10.1109/JBHI.2021.3119394>
104. Tian S, Wang M, Ma H, Huang P, Dai N, Sun Y, and Meng J. 2022b. Efficient tooth gingival margin line reconstruction via adversarial learning. *Biomedical Signal Processing and Control* 78:103954. <https://doi.org/10.1016/j.bspc.2022.103954>
105. Tran M. 2022. AI Isn't Science Fiction: It's Digital Design's Reality Deep learning proves effective in designing crowns. *Inside Dental Technology* 12.
106. Upmanyu S, Upmanyu A, Jamwal A, and Agrawal R. 2022. Machine Learning in CAD/CAM: What We Think We Know So Far and What We Don’t. In: Agrawal R, Jain JK, Yadav VS, Manupati VK, and Varela L, editors. Recent Advances in Industrial Production. Singapore: Springer Singapore. p 495-507.
107. Uzun G. 2008. An Overview of Dental CAD/CAM Systems. *Biotechnology & Biotechnological Equipment* 22:530-535. 10.1080/13102818.2008.10817506
108. Wang C, Zhang R, Wei X, Wang L, Xu W, and Yao Q. 2023a. Machine learning-based automatic identification and diagnosis of dental caries and calculus using hyperspectral fluorescence imaging. *Photodiagnosis Photodyn Ther* 41:103217. 10.1016/j.pdpdt.2022.103217
109. Wang C, Zhang R, Wei X, Wang L, Xu W, and Yao Q. 2023b. Machine learning-based automatic identification and diagnosis of dental caries and calculus using hyperspectral fluorescence imaging. *Photodiagnosis and Photodynamic Therapy* 41:103217.
110. Wang F, Tang Q, Xi S, Liu R, and Niu L. 2020. Comparison and evaluation of the morphology of crowns generated by biogeneric design technique with CEREC chairside system. *PLoS One* 15:e0227050. 10.1371/journal.pone.0227050
111. Wang G, Wang S, Dong X, Zhang Y, and Shen W. 2023c. Recent progress in additive manufacturing of ceramic dental restorations. *Journal of Materials Research and Technology* 26:1028-1049. <https://doi.org/10.1016/j.jmrt.2023.07.257>
112. Ward L, Agrawal A, Choudhary A, and Wolverton C. 2016. A general-purpose machine learning framework for predicting properties of inorganic materials. *npj Computational Materials* 2:16028. 10.1038/npjcompumats.2016.28
113. Watanabe H, Fellows C, and An H. 2022. Digital Technologies for Restorative Dentistry. *Dent Clin North Am* 66:567-590. 10.1016/j.cden.2022.05.006
114. World Dental Federation FDI. 2018. CAD/CAM Dentistry: Adopted by the FDI General Assembly: August 2017, Madrid, Spain. *Int Dent J* 68:18-19. 10.1111/idj.12373
115. Yamaguchi S, Lee C, Karaer O, Ban S, Mine A, and Imazato S. 2019. Predicting the Debonding of CAD/CAM Composite Resin Crowns with AI. *J Dent Res* 98:1234-1238. 10.1177/0022034519867641
116. Yang G, Ye Q, and Xia J. 2022a. Unbox the black-box for the medical explainable AI via multi-modal and multi-centre data fusion: A mini-review, two showcases and beyond. *Inf Fusion* 77:29-52. 10.1016/j.inffus.2021.07.016
117. Yang J, Liang Y, Zhang Y, Song W, Wang K, and He L. 2021. Exploring instance-level uncertainty for medical detection. Proceedings - International Symposium on Biomedical Imaging. p 448-452.
118. Yang L, and Shami A. 2020. On hyperparameter optimization of machine learning algorithms: Theory and practice. *Neurocomputing* 415:295-316. <https://doi.org/10.1016/j.neucom.2020.07.061>
119. Yang S, Lee H, Jang B, Kim KD, Kim J, Kim H, and Park W. 2022b. Development and Validation of a Visually Explainable Deep Learning Model for Classification of C-shaped Canals of the Mandibular Second Molars in Periapical and Panoramic Dental Radiographs. *Journal of Endodontics* 48:914-921. 10.1016/j.joen.2022.04.007
120. Zhang C, Fan L, Zhang S, Zhao J, and Gu Y. 2023a. Deep learning based dental implant failure prediction from periapical and panoramic films. *Quantitative Imaging in Medicine and Surgery* 13:935-945. 10.21037/qims-22-457
121. Zhang Y, Wang Y, Zhang Z, Wang Y, and Jia J. 2023b. Study on machine learning of molar incisor hypomineralization in an endemic fluorosis region in central China. *Front Physiol* 14:1088703. 10.3389/fphys.2023.1088703
122. Zhang Y, Weng Y, and Lund J. 2022. Applications of explainable artificial intelligence in diagnosis and surgery. *Diagnostics* 12:237.
